# Extracellular vesicles epitopes as potential biomarker candidates in patients with traumatic spinal cord injury

**DOI:** 10.3389/fimmu.2024.1478786

**Published:** 2024-11-27

**Authors:** Jason-Alexander Hörauf, Cora Rebecca Schindler, Inna Schaible, Minhong Wang, Birte Weber, André El Saman, Christiane Pallas, Marek Widera, Ingo Marzi, Dirk Henrich, Liudmila Leppik

**Affiliations:** ^1^ Goethe University Frankfurt, University Hospital, Department of Trauma Surgery and Orthopedics, Frankfurt, Germany; ^2^ Goethe University Frankfurt, University Hospital, Institute for Medical Virology, Frankfurt, Germany

**Keywords:** spinal cord injury, extracellular vesicles, biomarkers, epitopes, polytrauma

## Abstract

**Background:**

Extracellular vesicles (EVs), a heterogeneous group of cell-derived, membrane-enclosed vesicles bearing cell-specific epitopes, have been demonstrated to play a crucial role in neuronal-glial communication and the orchestration of neuroinflammatory processes. However, the existing evidence regarding their function as biomarkers and their role in the pathobiology of traumatic spinal cord injuries (tSCI), particularly in humans, is scarce.

**Objective:**

The primary goal of this study was to investigate whether a distinct pattern of EV surface epitopes detected in the plasma of individuals suffering from spinal cord injury is indicative of tSCI.

**Methods:**

The study includes patients with isolated tSCI (n=8), polytrauma patients without tSCI (PT; ISS ≥16, n=8), and healthy volunteers (HV; n=8). Plasma samples from tSCI and PT patients were collected right after admission to the emergency room (ER), 24 hours (24h), and 48h after trauma. EVs were isolated via size exclusion chromatography, and EVs’ surface epitopes were quantified with MACSPlex EV Kit Neuro (prototype product, Miltenyi Biotec) and compared among the groups. Additionally, results were correlated with clinical parameters.

**Results:**

In total, 19 epitopes differed significantly between the tSCI and the HV groups. Out of these 19, four (CD47, CD56, CD68, and ADAM17) were found to differ significantly among tSCI and PT groups. The expression of the CD47 epitope was found to correlate positively with the American Spinal Injury Association (ASIA) impairment scale.

**Conclusion:**

We identified four potential EV-based tSCI biomarkers (CD47+, CD56+, CD68+, and ADAM17+ EVs) that differ in tSCI, with CD47+ EVs showing a strong correlation with the neurological function in tSCI. Thus, future studies might further specify the relevance of potential tSCI-specific biomarkers and investigate underlying mechanisms of tSCI.

## 1 Introduction

Traumatic spinal cord injury (tSCI) represents a significant traumatic incident, impacting an estimated 250,000–500,000 new cases globally annually according to the World Health Organization (1). Moreover, tSCI is a significant health problem worldwide that not only dramatically affects individual life trajectories but also has a significant economic impact on the affected individual and the healthcare system, as treatment costs are high, and the affected individuals often require lifelong medical support (2).

Currently, the prevailing hypothesis of tSCI posits a dual-phase mechanism consisting of primary and secondary damage processes. The primary injury mechanism entails alterations in the spinal cord’s internal structure due to intense external forces and disruption of the blood-spinal cord barrier (BSCB) (3). Numerous studies have demonstrated that the primary injury mechanism can trigger a cascade of secondary damages including ischemia, demyelination, apoptosis, neuronal necrosis, and inflammation (4). This distinct acute (neuro-) proinflammatory response to the primary traumatic tSCI induces a reactive process of secondary damage also in the tissues surrounding the initial injury site, thus aggravating the neuronal damage and consequently the neurological dysfunctions (4). This acute phase response in the context of central nervous system (CNS) injury appears to play a central role in modulating secondary injury (5–7). Yet, owing to the complex nature of its pathophysiological processes, no discernible and efficient therapies targeting specific mechanisms or strategies aimed at promoting functional recovery have been identified for tSCI and repair of the damaged spinal cord remains a major clinical challenge.

In addition to the pathophysiological and therapeutic aspects, the identification of biomarkers is also significant for early diagnosis. Typical biomarkers such as glial fibrillary acidic protein (GFAP), neurofilaments, cleaved tau, myelin basic protein, neuron-specific enolase (NSE), and S100b are elevated after traumatic spinal cord injuries (8). However, it has been shown that NSE and S100b can also be elevated in the context of polytrauma, even without a tSCI (9, 10). Consequently, it is suggested that biomarkers be used to assess injury severity and treatment response rather than for diagnosis, due to their low specificity (11).

Recent findings indicate that extracellular vesicles (EVs), small membrane-bound nanoparticles, serve as pathological mediators in neurotrauma, exerting their effects not only within the central nervous system but also through interactions with the periphery (12, 13). EVs have the same topology as their original cell and promote intercellular communication, a function closely linked to their cargo, which includes various coding and non-coding RNAs (e.g., microRNAs), lipids, and proteins, in addition to their surface epitopes that all might contribute to pathophysiology upon tSCI (14). Since EVs have been shown to be present in numerous biological fluids, including blood (15), and cerebrospinal fluid (16), they are a promising diagnostic tool. Furthermore, EVs demonstrate the capacity to traverse the BSCB, a feat that nearly 98% of systemic drugs are unable to accomplish (17). Due to these capabilities, integration of EVs in the context of tSCI is gaining recognition as a promising therapeutic strategy (18). However, already in 2019, Yates et al. critically noted in their review that compared to brain pathologies, the role of EVs after tSCI has been somewhat overlooked. They emphasized that no data, particularly in humans, describes changes in the circulating EV populations and their impact on the pathophysiology of tSCI (19).

Thus, the main objectives of the present study were to compare the repertoire of EVs in plasma from patients with isolated tSCI, severely injured patients without SCI, and healthy controls by analyzing EV surface epitopes, and to identify potential SCI-specific EV biomarkers that enhance understanding of the complex acute phase response following tSCI.

## 2 Materials and methods

### 2.1 Study design

The Local Ethics Committee at the University of Frankfurt provided ethical approval for all studies (approval IDs 89/19; 375/14), which were carried out in compliance with the Declaration of Helsinki and the STROBE principles (20). Written informed consent was obtained from enrolled patients or their legally authorized representatives. This study includes trauma patients admitted to the Level 1 Trauma Center of the Frankfurt University Hospital (Frankfurt am Main, Germany) between 2016 and 2022. Blood samples from trauma patients were collected in 7.5 mL/2.7 mL tubes (S-Monovette^©^, Sarstedt Inc., Nümbrecht, NRW, Germany) containing 1.6 mg EDTA K at the time of admission to the emergency room (ER), 24, and 48 hours later, immediately stored on ice, and plasma was then collected by centrifugation for 15 minutes at 3500x g and 4°C. After centrifugation, the plasma samples were aliquoted and stored at -80°C until further analysis. Plasma samples from the healthy volunteers, serving as the control group, underwent the same processing as the patients’ samples.

This study comprises patients with isolated traumatic spinal cord injury (tSCI, n=8), polytrauma patients without SCI and TBI (PT, ISS ≥16, n=8), and healthy volunteers (n=8). Patients’ demographic and clinical characteristics are shown in **Table 1**. Exclusion criteria were previously known chronic, systemic inflammatory or metabolic syndrome, polyneuropathy, critical illness syndrome, neurodegenerative diseases (e.g., dementia, Parkinson’s disease), chronical alcohol abuse, organic brain syndromes (e.g., epilepsy, schizophrenia), stroke, post-traumatic resuscitation, minor age < 18 years, and sepsis.

**Table 1:** Demographic and clinical characteristics of patients.

	tSCI (n=8)	PT (n=8)	p-value
Male [number]	6	7	0.5218
Age [years] ± SD	48 ± 21.98	40 ± 13.79	0.2421
ISS [points] ± SD	25.38 ± 1.06	37.88 ± 15.76	**0.0003**
IL-6 ER [pg/ml] ± SD	17.56 ± 12.2	293.4 ± 141.7	**0.0006**
Leukocytes ER [/nl] ± SD	7.41 ± 2.17	11.77 ± 2.73	**0.0059**
Hemoglobin ER [g/dl] ± SD	12.36 ± 1.74	10.63 ± 2.52	0.1605
Lactate ER [mg/dl] ± SD	20.88 ± 10.26	29.38 ± 22.47	0.4563
LDH ER [U/l] ± SD	300.4 ± 146.5	475.6 ± 170.9	0.0830
Bilirubin ER [mg/dl] ± SD	0.35 ± 0.18	0.44 ± 0.28	0.6239
ICU/IMC stay [days] ± SD	8.8 ± 6.7	14.8 ± 9.9	0.1781
Hospital stay [days] ± SD	14.8 ± 10.6	35.3 ± 21.4	**0.0293**

ER, Emergency Room; ICU, Intensive Care Unit; IL, Interleukin; IMC, Intermediate Care; ISS, Injury Severity Score; PT, Polytrauma; tSCI, traumatic Spinal Cord Injury. The p values below or equal to 0.05 are marked in bold.

Neurological impairment was assessed after surgical treatment and clinical stabilization of the patient in the Intensive Care Unit (ICU) by an experienced neurology specialist based on the American Spinal Injury Association (ASIA) Impairment Scale (21). Briefly, ASIA scale assesses the patient’s sensory and motor functions and assigns the degree of injury into five grades from A (complete injury - no motor or sensory function below the level of injury) to E (normal function - both motor and sensory functions are intact).

### 2.2 EV isolation and characterization

Extracellular vesicles were isolated from 100 µl of plasma by size exclusion chromatography (Exo-Spin™, Cat. No. EX03, Cell Guidance Systems, Cambridge, UK) according to the manufacturer’s instructions. Briefly, the plasma was centrifuged at 16,000× g for 30 minutes at 4°C prior to EV isolation. Meanwhile, the Exo-Spin™ columns were equilibrated for 15 minutes at room temperature. The outlet plugs were then removed, the columns inserted into waste collection tubes, and the buffer inside the columns was aspirated and discarded. The columns were immediately washed twice with 250 µL of filtered (0.22 µM, Millex^®^ polyethersulfone syringe filter, Cat. No. SLGPR33RS, Merck, Darmstadt, Germany) phosphate-buffered saline (PBS) (1xDPBS, Cat. No. 14190169, ThermoFischer Scientific, MA USA). Subsequently, 100 µL of the prepared plasma samples were loaded onto the column. EVs were eluted from the columns with 180 µL of filtered PBS into a fresh collection tube. The collected EVs were briefly centrifuged at 100× g, aliquoted, and either directly analyzed using MACSPlex or frozen at −80°C until further analysis.

The number and size distribution of EV particles were determined by nanoparticle tracking analysis (NTA) (Nanosight NS500, Malvern Panalytical, Kassel, Germany), as described before (22). Protein concentration was measured by Coomassie Plus (Bradford) Assay (Cat. No. 23236, Thermo Fisher Scientific, Rockford, IL, USA). EV expression of CD63, CD9, and CD81 was confirmed by means of western blot analysis, as described before (23). 20µg of protein equivalent of EVs and 5µl Precision Plus Protein WesternC Standards (Cat. No. 161-0376, Bio-Rad, Feldkirchen, Germany) were gel-separated and first the antibody against CD81 (1:1000, Cat. No. 10630D, Invitrogen, Rockford, IL, USA), CD63 (1:1000, Cat. No. 10628D, Invitrogen) and CD9 (1:1000, Cat. No. 10626D, Invitrogen), and then horseradish peroxidase-linked antibody (1:2000, Cat. No. 7076, Cell Signaling Technology, Leiden, The Netherlands) were used. Transmission electron microscopy (TEM) staining was carried out in accordance with standard diagnostic procedures as described elsewhere (22). TEM analysis was performed with a EM900 TEM (Zeiss) instrument.

### 2.3 MACSPlex analysis

The bead-based multiplex exosome flow cytometry assay, MACSPlex EV Kit Neuro (prototype product^1^, Miltenyi Biotec, Bergisch Gladbach, Germany), was used to analyze the EV surface antigens as described previously (24). The prototype kit contained two sets of capture beads, each coated with distinct monoclonal antibodies targeting 39 EV surface antigens (panel A) and 22 EV surface antigens (panel B). A complete list of the bead populations can be found in **Supplementary Table S1**. Isolated EVs (20µg protein) from each sample (tSCI n=8, PT and healthy control groups, n=8), were first incubated with surface epitope-specific antibodies coupled with fluorescent-labeled beads (Panel A or B) and then incubated with MACSPlex Exosome Detect Reagents CD9, CD63 and CD81 (Cat. No. 130-108-813, Miltenyi Biotec), according to the manufacturer’s instructions and analyzed via flow cytometry (BD FACSCanto II, FACS DIVA Software, Heidelberg, Germany) (**Supplementary Figure S1**). For each sample, the resulting APC-A values were normalized to the mean APC-A values of the total amount of EVs measured by CD63, CD81, and CD9, and then the group mean was calculated and compared between each group.

### 2.4 Enzyme-linked immunosorbent assay for CD47

CD47 EV expression was measured via human CD47 ELISA Kit (A313572, Antibody.com, Stockholm, Sweden) in 40µl of each EV isolate. The obtained value for each sample was normalized for protein concentration and used to calculate the group mean.

### 2.5 Statistical analysis

The statistical software GraphPad Prism 10 (Dotmatics, San Diego, CA, USA) was used for all statistical analyses performed in this work. The values are presented as mean ± standard deviation (SD). Data were analyzed using the Kruskal–Wallis test followed by Dunn’s multiple comparison test. In order to determine correlations between EV epitope expression and injury characteristics, linear correlation analysis was performed using Spearman’s test [correlation coefficient ρ (rho)]. Results of EV surface epitope expression analysis are presented as box plots of the median in diagrams. Results were considered statistically significant when p ≤0.05.

## 3 Results

### 3.1 Patients clinical characteristics

Overall, eight patients with isolated traumatic SCI (tSCI group) and eight polytrauma patients (without tSCI, PT group) admitted to the ER met the inclusion criteria and were enrolled in the study. The demographic and clinical characteristics of the patients are shown in **Table 1**. Eight healthy volunteers were recruited representing healthy controls. In both patient groups, the majority of patients were male and the patients in the tSCI group were slightly older (48 years vs. 40 years; **Table 1**). The mean Injury Severity Score (ISS), Interleukin (IL)-6 measured in the ER, and the number of leukocytes were significantly lower in the tSCI group. There were no significant differences in the other laboratory parameters (hemoglobin, lactate, lactate dehydrogenase, bilirubin) among the groups. Regarding the length of stay at the ICU/IMC, there was no significant difference between the tSCI and PT groups, however the total length of hospitalization was significantly lower in the tSCI group. Regarding the neurological status of patients with traumatic spinal cord injuries, four cases were classified as ASIA grade A, one as grade B, and three as grade C. Six of the injuries were located in the cervical spine and two in the lumbar spine.

### 3.2 EVs surface epitope expression

**Figure 1:**
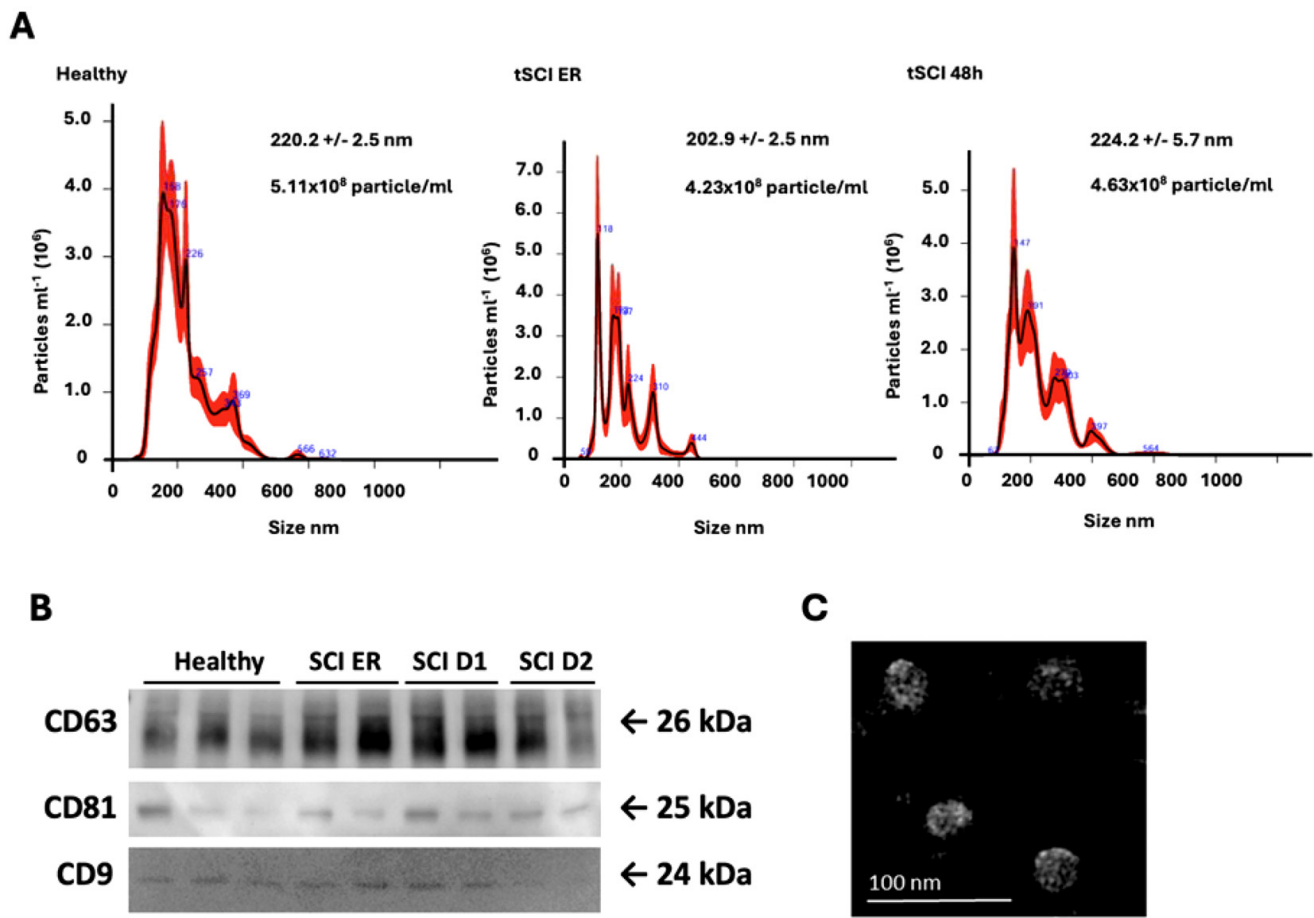
Characterization of plasma-isolated Extracellular Vesicles (EVs) **(A)** Representative results of Nanoparticle Tracking Analysis (NTA) conducted on EV isolates (1 in 100 dilution) from healthy volunteers, patients with traumatic Spinal Cord Injury (tSCI) at timepoints Emergency Room (ER) admission and 48 hours (h) after trauma. The mean particle size (nm) and concentration (particle/ml) are indicated in the graphs. **(B)** Representative Western-blot analysis of CD63, CD81, and CD9 expression in EVs collected from healthy volunteers and tSCI patients at ER admission, 24h and 48h after trauma. **(C)** Representative transmission electron microscopy-image of healthy volunteer EV isolate. Scale bar = 100 nm.

To compare EV surface epitopes between the patient cohorts and healthy controls, we isolated and characterized EVs via NTA analysis (**Figure 1A**). The EV expression of CD63, CD81 and, CD9 was confirmed by means of western blot (**Figure 1B**), and the size and overall morphology of the isolated particles were evaluated with TEM (**Figure 1C**; **Supplementary Figure S2**).

To identify tSCI- specific EVs we first performed a comparison of EV-epitope expression in control EVs and EVs isolated at different time points from tSCI patients (ER and 48h). Out of 61 analyzed EV epitopes, significant differences in the expression of 19 epitopes were found between the tSCI patients’ group and the HC group (**Table 2**).

**Table 2:** EV- surface epitopes with differential expression in tSCI and healthy controls.

Epitope	Time point	Control, APC median ± SD	tSCI, APC median ± SD	Regulation	p-value
CD133	ER	0.798 (± 0.336)	0.390 (± 0.126)	down	0.0085
CD45	ER	0.719 (± 0.127)	0.457 (± 0.083)	down	0.0101
CD90	ER	0.669 (± 0.183)	0.404 (± 0.234)	down	0.0494
CD36	ER	4.664 (± 2.224)	8.092 (± 2.402)	up	0.0343
CD68	ER	1.784 (± 0.796)	0.879 (± 0.346)	down	0.0340
CX3CR1	ER	0.928 (± 0.439)	0.487 (± 0.186)	down	0.0281
CD196	ER	1.138 (± 0.507)	0.336 (± 0.224)	down	0.0093
CD44	24h	0.440 (± 0.072)	0.625 (± 0.170)	up	0.0404
GFAP	24h	0.850 (± 0.170)	1.436 (± 0.528)	up	0.0070
CD171	24h	0.369 (± 0.040)	0.564 (± 0.146)	up	0.0167
CD45RB	24h	0.697 (± 0.093)	1.229 (± 0.380)	up	0.0167
CD64	24h	0.292 (± 0.115)	0.629 (± 0.286)	up	0.0070
CD56	24h 48h	0.657 (± 0.140)	1.074 (± 0.474) 0.905 (± 0.240)	up	0.0499 0.0281
CD29	ER 48h	1.523 (± 0.397)	3.692 (± 2.818) 2.666 (± 0.896)	up	0.0211 0.0226
CD13	ER 48h	1.026 (± 0.306)	0.517 (± 0.141) 0.674 (± 0.148)	down	0.0021 0.0281
MBP	ER 48h	0.780 (± 0.432)	0.276 (± 0.123) 0.381 (± 0.264)	down	0.0073 0.0459
ADAM17	ER 48h	1.382 (± 0.497)	0.555 (± 0.288) 0.732 (± 0.494)	down	0.0038 0.0197
CD31	48h	0.558 (± 0.152)	1.067 (± 0.341)	up	0.0104
CD47	48h	1.006 (± 0.169)	1.648 (± 0.419)	up	0.0038

ADAM17, A disintegrin and metalloprotease 17; CX3CR1, CX3C motif chemokine receptor 1; GFAP, Glial fibrillary acidic protein; MBP, Myelin Basic Protein; tSCI, traumatic Spinal Cord Injury.

At the ER, the expressions of CD133+, CD45+, CD90+, CD68+, CX3CR1+, and CD196+ EVs were significantly downregulated in the tSCI group, whereas the expression of CD36+ EV was up-regulated. CD44+, GFAP+, CD171+, CD45RB+, and CD64+ EVs were all significantly upregulated 24h after tSCI compared to the healthy control group. Out of five EV epitopes differentially presented at the two time-points of analysis, the expression of CD29 and CD56 was upregulated in the tSCI group, while CD13, MBP, and ADAM17 were downregulated compared to control (**Table 2**). The expressions of CD31+ and CD47+ EVs were significantly upregulated at 48h time point only.

**Figure 2:**
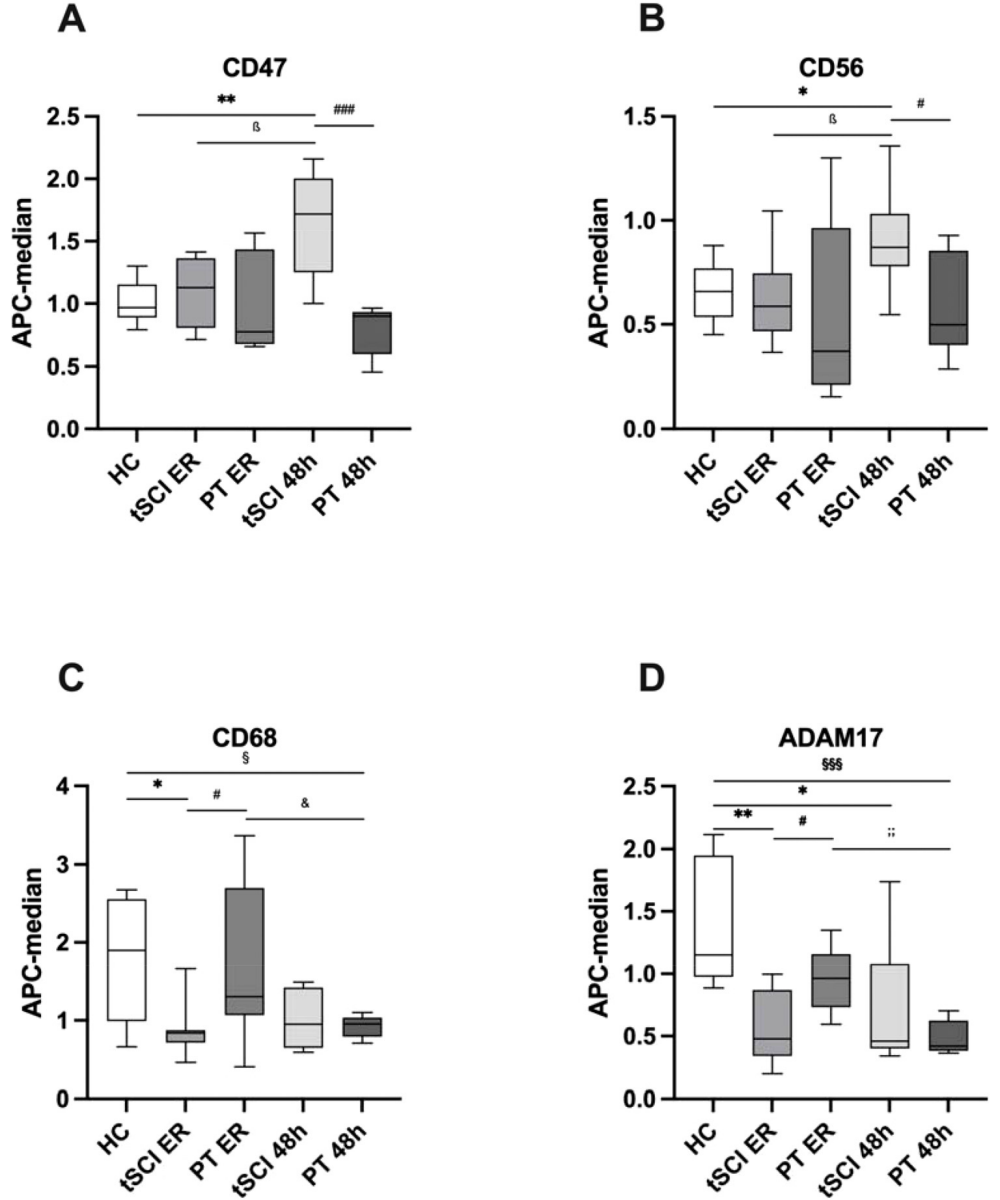
Extracellular Vesicle (EV) surface expression pattern between healthy control (HC) subjects and patients with isolated traumatic spinal cord injury (tSCI) and polytrauma patients (PT) without tSCI at the time points of emergency room (ER) admission and 48 hours (h) after trauma. **(A)** CD47+ EVs were significantly increased in tSCI patients 48h after trauma compared to HC and PT 48h groups. **(B)** CD56+ EVs were significantly increased in tSCI patients 48h after trauma compared to HC and PT 48h groups. **(C)** CD68+ EVs were significantly decreased in tSCI patients at ER admission compared to HC and PT ER groups. **(D)** ADAM17+ EVs were significantly decreased in tSCI patients at ER admission compared to HC and PT ER groups. Results are shown as boxplots of the median. *, ß, #, &, § p< 0.05; **;; p< 0.01; §§§; p<0.001 among marked groups. .

Next, we analyzed which of the 19 identified trauma-related EVs were specific for tSCI. Through a comparative analysis between EV isolates from the tSCI and PT groups, we identified six EV fractions with expression levels significantly altered exclusively in tSCI patients (**Figure 2**).

Specifically, the amount of CD47+ EVs was significantly higher in the tSCI group at 48h compared to both the healthy control group (**Table 2**) and the PT group at 48h (p= 0.0003) (**Figure 2A**). In addition, there was a significant increase (p= 0.0207) in CD47+ EVs in the tSCI group between the ER and 48h.

At 48h post-trauma in the tSCI group, CD56+ EVs were significantly increased compared to the healthy control group and the PT group 48h after trauma (p= 0.0148) (**Figure 2B**). Furthermore, there was a significant increase in CD56+ EVs within the tSCI group (tSCI ER: 0.622 ± 0.215 vs. tSCI 48h; p= 0.0207).

CD68+ EVs were significantly decreased in the tSCI group at the ER compared to the healthy control group and the PT group at the same time point (p= 0.0274) (**Figure 2C**). Moreover, in the PT group, CD68+ EVs were also significantly decreased at the 48h time point compared to the healthy control group (p= 0.0379) and PT group at the ER (p= 0.0360).

In the tSCI group at the time of ER admission, ADAM17+ EVs were significantly decreased compared to both the healthy control group and the PT group at the same time point (PT ER: 0.954 ± 0.253; p= 0.0148) (**Figure 2D**). Furthermore, ADAM17+ EVs were significantly decreased at 48h post-trauma in the tSCI group (tSCI 48h: 0.732 ± 0.494; p= 0.0148) as well as in the PT group (PT 48h: 0.499 ± 0.134; p= 0.0003) compared to the healthy control group. Additionally, there was a significant decrease in ADAM17+ EVs within the PT group (PT ER vs. PT 48h; p= 0.0012).

**Figure 3:**
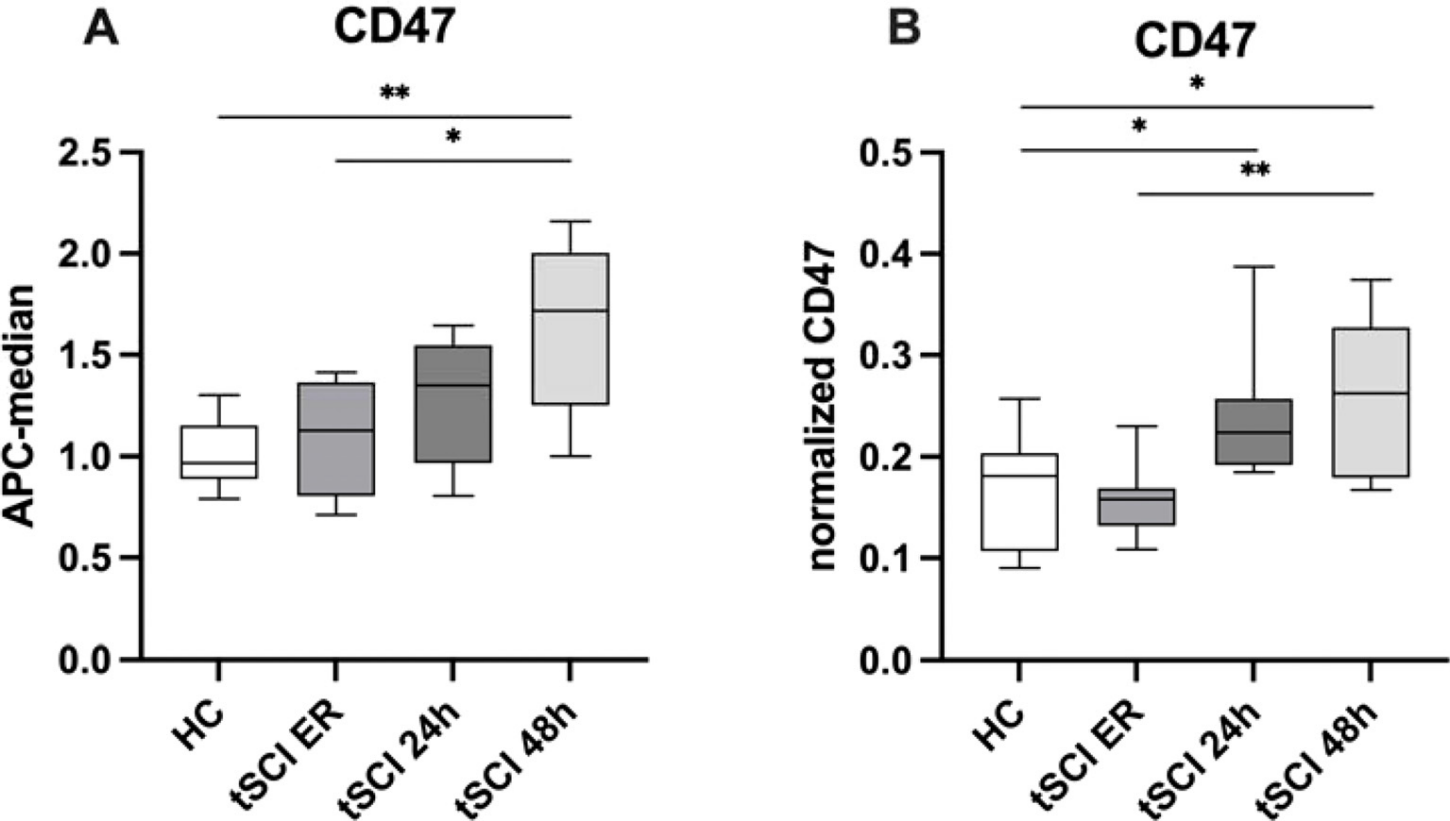
Validation of CD47 EV expression pattern in patients with isolated traumatic spinal cord injury (tSCI) at the time points of ER, 24h and, 48h after trauma. **(A)** CD47+ EVs were significantly increasing in tSCI patients over the time points. **(B)** CD47 ELISA analysis in HC and tSCI ER, 24h and 48h -EV isolates. CD47 was measured in EV isolates (n=8) via ELISA, normalized to EV amount (protein amount) of the same samples and the relative signal intensity values are shown in the figure. Results are shown as boxplots of the median. * p< 0.05; ** p< 0.01 among marked groups.

To further validate the temporary increase of tSCI-specific CD47+ EVs, we quantified the amount of these EVs using MACSPlex analysis and ELISA in plasma EV isolates obtained from tSCI patients at ER, 24h, and 48h time points (**Figure 3**).

The findings confirmed an increase in CD47+ EVs over time in patients after tSCI. Moreover, the results of ELISA further demonstrated a significant up-regulation of CD47+ EVs in tSCI patients compared to healthy controls, and a significant increase of these EVs within the tSCI group over the respective time points.

### 3.3 Correlation between EV surface epitopes expression and neurological status

To assess the diagnostic and prognostic potential of the identified differentially expressed EV epitopes, a correlation analysis with neurological impairment assessed by ASIA grade was conducted. A strong positive correlation (rho= 0.7715, p= 0.0429) was found between CD47+ EVs and the ASIA grade, indicating that poorer neurological status after trauma is associated with higher expression of CD47+ EVs. CD56+ EVs showed a positive trend, while CD68+ and ADAM17+ EVs both showed a negative trend with the neurological impairment of the patients; however, these findings were not statistically significant (**Table 3**).

**Table 3:** Correlation analysis of Extracellular Vesicle (EV) surface epitope expression and neurological impairment assessed by American Spinal Injury Association (ASIA) Impairment Scale.

EV marker at ER	Spearman's ρ (rho)	p-value
CD47	0.7715	**0.0429**
CD56	0.6429	0.0952
CD68	-0.5915	0.1381
ADAM17	-0.6429	0.0952

ADAM, A Disintegrin And Metalloprotease; CD, Cluster of Differentiation; ER, Emergency Room; p = ≤0.05.

The p values below or equal to 0.05 are marked in bold.

## 4 Discussion

EVs play crucial roles in neural plasticity, neuro-glia communication, and immune response throughout disease conditions due to their varied biological cargo and surface epitopes (12). However, our understanding of the pathophysiological functions of EVs in the CNS remains limited. Since the composition of EVs, including their cargo and surface epitopes, may reflect cell origin and activity, the analysis of EV surface signatures in plasma from trauma patients has the capability of revealing characteristic tSCI-related changes in the EV population. Here, we examined the composition of surface epitopes in EVs isolated from the plasma of healthy controls and two groups of trauma patients - those with isolated tSCI and those with polytrauma (without tSCI).

### 4.1 Trauma-related EVs

We observed that 19 EV-epitopes (**Figure 2**, **Table 2**) were expressed differentially among patients and healthy controls. Out of these 19, four were found to be tSCI- specific, whereas the rest appeared to be trauma-related EVs.

Depending on the time point considered (ER, 24h, 48h), a distinct pattern in the upregulation or downregulation of the analyzed EV populations emerged. For instance, at the ER time point, most significantly altered EVs were downregulated, while at 24 and 48 hours after trauma all significantly changed EVs were upregulated. Among the EVs altered at two time points, the findings were heterogeneous (both, up- and down- regulated). Consistently, some studies revealed that immediately after trauma, the amount of EV particles significantly increased in the context of an induced post-traumatic immune response (25, 26), whereas other studies observed no significant changes or even significantly reduced particle amounts (27, 28), suggesting a dynamic, possibly trauma type- or trauma severity-dependent change in EV populations (13). In line with this, it has been demonstrated that endothelial cells release qualitatively and quantitatively different EVs with varying protein content depending on the stimuli they were exposed to (29, 30). In this study, the APC-A values for each EV epitope were normalized to the mean APC-A values of CD63, CD81, and CD9 (representing total EVs), allowing us to compare the proportion of specific EV fractions across the groups. This method reduces the dependence on the total number of EVs, as it focuses on the relative expression of specific epitopes rather than the absolute EV quantity.

It is also possible that trauma-induced internalization of EVs by various recipient cells, such as macrophages or endothelial cells, in the context of remote cell-to-cell communication, could cause these EV epitope changes, leading to alterations in both the functional and phenotypic characteristics of the target cells (31). Furthermore, trauma-induced metabolic changes may also influence EV biogenesis. For example, lactate levels were inversely correlated with platelet-derived microparticle levels, suggesting a plausible mechanism for decreased particle formation in severely injured trauma patients (32). Therefore, it is also conceivable that various cell types might be influenced differently under trauma-induced altered conditions in their generation of EVs, leading to variations in EV surface epitope profiles. Additionally, other non-trauma-associated confounding factors, such as patient age, pre-existing conditions like alcohol abuse, or ongoing medication, appear to influence the post-traumatic inflammatory response, which may also affect the biogenesis of EVs, including their surface profile and cargo upon trauma (33).

It is possible a complex and dynamic EV response occurs following trauma, given the cellular and humoral intricacies of secondary injury after tSCI. One of the early responders to tSCI, besides the local neural tissue cells (neurons, astrocytes, oligodendrocytes), are resident microglia cells, followed by infiltrating neutrophils who peak at approximately 24 hours post-SCI (34). In contrast to neutrophils, lymphocytes peak significantly later and weaker (34), suggesting that cell responses to tSCI involve both temporal and spatial aspects (35). Similarly, cytokine/chemokine regulation/expression after trauma follows a temporal pattern (36). While trauma to the spinal cord leads to a significant upregulation of IL-1β, TNFα, and IL-6 early after injury, anti-inflammatory cytokines like IL-10 or TGF-β1 lag further behind, peaking around 3-7 days post-injury (37). Since EVs can trigger both pro-inflammatory and anti-inflammatory responses depending on the clinical scenario, trauma may also lead to a differential upregulation and downregulation of EVs with both pro-inflammatory and anti-inflammatory properties, thereby modifying the course of the acute phase of secondary damage. The differentially expressed EVs we observed at various time points underscore the complex influence of EVs on the post-traumatic inflammatory response.

### 4.2 tSCI-specific EVs

Out of the four EV populations which exhibited specificity for tSCI (CD47+, CD56+, CD68+, and ADAM17+), CD68+ and ADAM17+ EVs were downregulated at the ER time point, while CD47+ and CD56+ EVs were increased at the 48-hour time point.

Our results indicate that CD47+ EVs exhibited temporary increase following trauma. CD47 (integrin-associated protein, IAP) is a glycoprotein expressed on a variety of cell types including hematopoietic cells as well as neuronal and lymphoid tissues (38). Under physiological conditions, neuronal-expressed CD47 binds to its receptor signal-regulatory protein alpha (SIRPα) expressed on macrophages/monocytes that provide a ‘don’t eat me’ signal, and protect healthy neurons from unintentional engulfment and elimination by those immune cells (39). However, in the context of neuroinflammatory events such as SCI, this SIRPα-CD47 interaction can prevent rapid clearance of degenerated myelin necessary for axon regeneration (40, 41), indicating that CD47 and SIRPα may have a dual role in certain pathological conditions such as neuroinflammation (42). Moreover, it was shown that CD47 on exosomes provides immune escape (43). A study by Zhuang et al. revealed significantly elevated expression of CD47 exosomes derived from patients with Diffuse large B cell lymphoma (DLBCL), suggesting a mechanism by which DLBCL cells escape immune-mediated clearance by phagocytes (44). Our data show that CD47+ EV level correlate positively with the ASIA impairment score in tSCI patients, suggesting the possible involvement of the CD47-SIRPα inhibitory mechanism in preventing axonal regeneration after tSCI. However, given the small sample size, the statistical power of the correlation analysis should be interpreted with caution. These findings need to be validated in larger patient cohorts. Due to the rarity of traumatic spinal cord injuries, multi-center studies are essential to include a sufficient number of patients. Additionally, studies with longer follow-up periods are needed to further explore the relationship between surface marker expression and neurological outcomes. Based on the literature and our current findings, CD47+ EVs may not only serve as a potential biomarker but also as a promising therapeutic target for promoting neuronal regeneration following traumatic spinal cord injury. Despite the possibility that CD47+ EVs come from non-neural tissue cells because CD47 is expressed on a wide range of cell types, the lack of these EVs’ presence in polytrauma patients suggests that they have a neuronal origin.

In addition to CD47+ EVs, CD56+ EVs were also increased at the 48-hour time point in tSCI patients. The role of CD56+ EV after tSCI could be different depending on the cellular origin of these EVs. CD56 (Neural Cell Adhesion Molecule 1, NCAM-1) is a well-known marker of natural killer (NK) cells, which produce immunoregulatory cytokines (IFN-γ, TNF-α) and exhibit cytotoxic activity (45). The significant increase of NK cells with an activated phenotype was previously shown in blood samples 24h post tSCI (46). The Important role of CD56 molecule in NK-promoted inflammation and cytotoxicity was demonstrated in knockout experiments (47, 48) and, moreover, the ability of NK-originated CD56+ exosome to prime naive T cells (49) and target cells for attack (50) was described. Therefore, the increase in CD56+ EVs observed in our study may reflect an NK cell-mediated inflammatory acute phase of secondary injury. However, given that impairment of NK cells in the subacute and chronic phases of tSCI, contributing to the development of Spinal Cord Injury-Induced Immunodeficiency Syndrome (SCI-IDS) (51), has been previously described (52), the increase of CD56+EVs may also imply time-dependent changes in the secretion of CD56+ EVs after tSCI.

However, CD56 is not only a marker for NK cells but is also expressed in neural tissue, where it was originally identified (53). NCAM expression and distribution were shown to change dynamically after tSCI, with an initial decrease at the lesion site and later an increase in the injury center-surround (54). Moreover, important roles of NCAM in axon regeneration, synaptogenesis, apoptosis inhibition, and thereby locomotor recovery after tSCI were shown in experiments with NCAM-deficient mice (54). Recently, exosomes derived from a CD271+CD56+ bone marrow mesenchymal stem cell (BMSC) subpopulation were found to promote axon regeneration and functional recovery after tSCI in mice (55). Thus, the role of CD56+ EVs, whether beneficial or disadvantageous for tSCI outcomes, may depend on the origin of the EV-parent cell (neural tissue, BMSCs origin, or NK cells). Further investigations are necessary to better understand and differentiate the impact of the respective cell entities and their EVs on the injury response in acute tSCI.

In contrast to CD47+ and CD56+ EVs, CD68+ EVs were significantly reduced in tSCI patients upon admission to the ER. CD68, a widely recognized macrophage-specific surface marker (56), is also expressed in the lysosomal membrane of microglia (57) and on the surface of human microglial-derived extracellular vesicles (M-EVs) (58). Microglia are the resident immune cells of the CNS, playing a crucial role in maintaining homeostasis, as they are the primary source of inflammatory mediators in the CNS (59). Recent studies suggest a beneficial role of M-EVs under neuroinflammatory conditions, as M-EVs mediate enhanced autophagy activation in other microglial cells, preventing the accumulation of (neuro-) toxic molecules and dying cells to promote CNS homeostasis (60). Moreover, M-EVs were capable of inducing the recruitment and differentiation of oligodendrocyte progenitor (OPC) cells into myelin-forming cells, facilitating remyelination and protecting axons from degeneration (61). However, in contrast to these M-EVs produced by pro-regenerative microglia, M-EVs produced by inflammatory microglia inhibit OPC differentiation into myelin-forming cells, exerting opposite effects on remyelination. This indicates that M-EVs are multimodal and multitarget signaling molecules that exist in a continuum of different phenotypes and properties depending on the type of conditioning stimulus (61). Taking this into account, trauma-induced SCI CD68+ EV downregulation in the acute phase may promote a pro-inflammatory environment that inhibits beneficial regenerative processes, thus contributing to secondary spinal cord damage. Since we did not observe a downregulation of CD68+ EVs at the ER time point in polytrauma patients, this might be a neurotrauma-specific pathomechanism during the very early acute phase of SCI.

We can only speculate whether the measured CD68+ EVs are actually of microglial origin or if they might instead be from blood-derived macrophages. After SCI, monocyte/macrophage blood levels peak between 3-7 days post-SCI and remain elevated for several months thereafter (37). Moreover, immunohistochemistry of the injured spinal cord in a contusive SCI rat model revealed a significant presence of CD68+/CD163- macrophages from the third day onwards (62). Since significant monocyte/macrophage infiltration occurs no earlier than three days after the injury, CD68+ EVs may initially remain downregulated and only become significantly upregulated a few days later. The significant difference between tSCI and PT patients may be due to the markedly higher injury severity in PT patients, as the extent of injury severity correlates linearly with the expression of other inflammatory mediators such as Monocyte Chemoattractant Protein-1 (MCP-1), a key driver in promoting monocyte infiltration to the site of injury (63).

Similar to CD68+ EVs, ADAM17+ (A Disintegrin And Metalloprotease 17) EVs were significantly reduced in SCI patients upon admission to the emergency room. ADAM17 is a ubiquitously expressed metalloprotease enzyme involved in the proteolytic processing and release of cell surface molecules such as TNF-α, which are important for regulating inflammatory processes and cell-cell communication (64). Several studies have shown that ADAM17-induced signaling is vital for the survival of various cultured neuronal cell lines, as inhibition of ADAM17 resulted in upregulated apoptosis in microglia and oligodendrocytes (65, 66). Integrins, such as integrin β1 or β2, are upregulated in the nervous system after tSCI and seem to sustain neuroinflammatory processes, with several ADAMs, including ADAM17, known to interact with these integrins (67, 68). It has been shown that cellular integrin α5β1 and exosomal ADAM17 mediate the binding and uptake of these exosomes in cancer cells (69). Moreover, both α5β1 integrin and β1 integrin decrease ADAM17 enzymatic activity (70). The upregulation of various integrins in neural tissue may have led to increased uptake and thus systemic consumption of ADAM17+ EVs upon tSCI. The downregulation of ADAM17+ EVs might be a neurotrauma-specific pathomechanism during the very early acute phase of SCI, as we did not observe this effect in polytrauma patients.

There are limitations in this study that need to be addressed. Due to the relative rarity of isolated tSCIs, the sample size in this monocentric study with prospectively collected patient blood samples is very limited (n=8 per group). However, the patients included in the isolated tSCI group represent a homogeneous cohort without relevant pre-existing conditions or chronic medication. We must also point out that the polytrauma patients were significantly more severely injured, which may have an influence on the EV-related inflammation response. However, the differences compared to patients with isolated tSCIs seem to be relevant, as in those polytrauma patients all inflammatory mechanisms are mostly activated. In common with all trauma studies, the groups studied have a higher proportion of male patients, which precluded a sufficient investigation of a possible gender-specific influence on the EV-related post-traumatic inflammatory response (71, 72).

## 5 Conclusion

Taken together, we identified four potential EV-based tSCI biomarkers (CD47+, CD56+, CD68+, and ADAM17+ EVs). The expression of CD47+ EVs has been found to increase with time after injury and strongly correlate with the neurological outcome of tSCI patients. These findings suggest that CD47+ EVs may be a factor in the secondary injury that promotes neuroinflammation. Our work also highlights the complex and dynamic EV-mediated pathophysiological processes following tSCI. Based on these results, future studies need to further evaluate the specificity of these potential tSCI-specific biomarkers and investigate the mechanistic aspects of tSCI to reveal potential therapeutic targets for the treatment of tSCI.

## Data Availability

The original contributions presented in the study are included in the article/**Supplementary Material**. Further inquiries can be directed to the corresponding author.

## Footnotes

1 At the time the experiments were conducted, the MACSPlex EV Kit Neuro was available only as a prototype. In the meantime, Miltenyi Biotec has released the MACSPlex EV Kit Neuro as a commercially available product (Cat. No. 130-133-425), with some bead populations having been replaced.

## References

[1] Bickenbach J Officer A Shakespeare T von Groote P World Health Organization The International Spinal Cord Society. International perspectives on spinal cord injury/edited by Jerome Bickenbach … [et al]. Geneva: World Health Organization (2013). Available at: https://iris.who.int/handle/10665/94190.

[2] Malekzadeh H Golpayegani M Ghodsi Z Sadeghi-Naini M Asgardoon M Baigi V. Direct cost of illness for spinal cord injury: A systematic review. Global Spine J. (2022) 12:1267–81. doi: 10.1177/21925682211031190 PMC9210246 34289308

[3] Jin LY Li J Wang KF Xia WW Zhu ZQ Wang CR. Blood–spinal cord barrier in spinal cord injury: A review. J Neurotrauma. (2021) 38:1203–24. doi: 10.1089/neu.2020.7413 33292072

[4] Alizadeh A Dyck SM Karimi-Abdolrezaee S. Traumatic spinal cord injury: an overview of pathophysiology, models and acute injury mechanisms. Front Neurol. (2019) 10:282. doi: 10.3389/fneur.2019.00282 30967837 PMC6439316

[5] Schindler CR Lustenberger T. Focus on challenges and advances in the treatment of traumatic brain injury. Eur J Trauma Emerg Surg. (2024) 52:1307–12. doi: 10.1007/s00068-024-02623-7 39292240

[6] Ahuja CS Wilson JR Nori S Kotter MRN Druschel C Curt A. Traumatic spinal cord injury. Nat Rev Dis Primers. (2017) 3:17018. doi: 10.1038/nrdp.2017.18 28447605

[7] Aghili-Mehrizi S Williams E Yan S Willman M Willman J Lucke-Wold B. Secondary mechanisms of neurotrauma: A closer look at the evidence. Diseases. (2022) 10:30. doi: 10.3390/diseases10020030 35645251 PMC9149951

[8] Kwon BK Bloom O Wanner IB Curt A Schwab JM Fawcett J. Neurochemical biomarkers in spinal cord injury. Spinal Cord. (2019) 57:819–31. doi: 10.1038/s41393-019-0319-8 31273298

[9] Savola O Pyhtinen J Leino TK Siitonen S Niemelä O Hillbom M. Effects of head and extracranial injuries on serum protein S100B levels in trauma patients. J Trauma: Injury Infect Crit Care. (2004) 56:1229–34. doi: 10.1097/01.TA.0000096644.08735.72 15211130

[10] Pelinka LE Hertz H Mauritz W Harada N Jafarmadar M Albrecht M. Nonspecific increase of systemic neuron-specific enolase after trauma: clinical and experimental findings. Shock. (2005) 24:119–23. doi: 10.1097/01.shk.0000168876.68154.43 16044081

[11] Yue JK Winkler EA Rick JW Deng H Partow CP Upadhyayula PS. Update on critical care for acute spinal cord injury in the setting of polytrauma. Neurosurg Focus. (2017) 43:E19. doi: 10.3171/2017.7.FOCUS17396 29088951

[12] Dutta D Khan N Wu J Jay SM. Extracellular vesicles as an emerging frontier in spinal cord injury pathobiology and therapy. Trends Neurosci. (2021) 44:492–506. doi: 10.1016/j.tins.2021.01.003 33581883 PMC8159852

[13] Weber B Franz N Marzi I Henrich D Leppik L. Extracellular vesicles as mediators and markers of acute organ injury: current concepts. Eur J Trauma Emerg Surg. (2022) 48:1525–44. doi: 10.1007/s00068-021-01607-1 PMC7856451 33533957

[14] Pegtel DM Gould SJ. Exosomes. Annu Rev Biochem. (2019) 88:487–514. doi: 10.1146/annurev-biochem-013118-111902 31220978

[15] Hazelton I Yates A Dale A Roodselaar J Akbar N Ruitenberg MJ. Exacerbation of acute traumatic brain injury by circulating extracellular vesicles. J Neurotrauma. (2018) 35:639–51. doi: 10.1089/neu.2017.5049 29149810

[16] Kuharić J Grabušić K Tokmadžić VS Štifter S Tulić K Shevchuk O. Severe traumatic brain injury induces early changes in the physical properties and protein composition of intracranial extracellular vesicles. J Neurotrauma. (2019) 36:190–200. doi: 10.1089/neu.2017.5515 29690821

[17] Ha D Yang N Nadithe V. Exosomes as therapeutic drug carriers and delivery vehicles across biological membranes: current perspectives and future challenges. Acta Pharm Sin B. (2016) 6:287–96. doi: 10.1016/j.apsb.2016.02.001 PMC4951582 27471669

[18] Shen Y Cai J. The importance of using exosome-loaded miRNA for the treatment of spinal cord injury. Mol Neurobiol. (2023) 60:447–59. doi: 10.1007/s12035-022-03088-8 PMC9849169 36279099

[19] Yates AG Anthony DC Ruitenberg MJ Couch Y. Systemic immune response to traumatic CNS injuries—Are extracellular vesicles the missing link? Front Immunol. (2019) 10:2723. doi: 10.3389/fimmu.2019.02723 31824504 PMC6879545

[20] von Elm E Altman DG Egger M Pocock SJ Gøtzsche PC Vandenbroucke JP. Strengthening the reporting of observational studies in epidemiology (STROBE) statement: guidelines for reporting observational studies. BMJ. (2007) 335:806–8. doi: 10.1136/bmj.39335.541782.AD PMC2034723 17947786

[21] Rupp R Biering-Sørensen F Burns SP Graves DE Guest J Jones L. International standards for neurological classification of spinal cord injury. Topics Spinal Cord Injury Rehabil. (2021) 27:1–22. doi: 10.46292/sci2702-1 PMC8152171 34108832

[22] Weber B Ritter A Han J Schaible I Sturm R Relja B. Development of a sampling and storage protocol of extracellular vesicles (EVs)—Establishment of the first EV biobank for polytraumatized patients. IJMS. (2024) 25:5645. doi: 10.3390/ijms25115645 38891833 PMC11172154

[23] Weber B Henrich D Schindler CR Marzi I Leppik L. Release of exosomes in polytraumatized patients: The injury pattern is reflected by the surface epitopes. Front Immunol. (2023) 14:1107150. doi: 10.3389/fimmu.2023.1107150 36969201 PMC10034046

[24] Schindler CR Hörauf JA Weber B Schaible I Marzi I Henrich D. Identification of novel blood-based extracellular vesicles biomarker candidates with potential specificity for traumatic brain injury in polytrauma patients. Front Immunol. (2024) 15:1347767. doi: 10.3389/fimmu.2024.1347767 38533491 PMC10963595

[25] Curry N Balvers K Kleinveld D Boïng A Nieuwland R Goslings J. Endogenous microparticles drive the proinflammatory host immune response in severely injured trauma patients. Crit Care. (2015) 19:P313, cc14393. doi: 10.1186/cc14393 25565646

[26] Kuravi SJ Yates CM Foster M Harrison P Hazeldine J Hampson P. Changes in the pattern of plasma extracellular vesicles after severe trauma. PloS One. (2017) 12:e0183640. doi: 10.1371/journal.pone.0183640 28837705 PMC5570308

[27] Matijevic N Wang YW Holcomb JB Kozar R Cardenas JC Wade CE. Microvesicle phenotypes are associated with transfusion requirements and mortality in subjects with severe injuries. J Extracell Vesicle. (2015) 4:29338. doi: 10.3402/jev.v4.29338 PMC4685295 26689982

[28] Caspers M Schäfer N Fröhlich M Bouillon B Mutschler M Bauerfeind U. Microparticles profiling in trauma patients: high level of microparticles induce activation of platelets in *vitro* . Eur J Trauma Emerg Surg. (2020) 46:43–51. doi: 10.1007/s00068-019-01111-7 30864053

[29] Jimenez JJ Jy W Mauro LM Soderland C Horstman LL Ahn YS. Endothelial cells release phenotypically and quantitatively distinct microparticles in activation and apoptosis. Thromb Res. (2003) 109:175–80. doi: 10.1016/S0049-3848(03)00064-1 12757771

[30] Peterson DB Sander T Kaul S Wakim BT Halligan B Twigger S. Comparative proteomic analysis of PAI-1 and TNF-alpha-derived endothelial microparticles. Proteomics. (2008) 8:2430–46. doi: 10.1002/pmic.200701029 PMC4753841 18563738

[31] Ratajczak J Miekus K Kucia M Zhang J Reca R Dvorak P. Embryonic stem cell-derived microvesicles reprogram hematopoietic progenitors: evidence for horizontal transfer of mRNA and protein delivery. Leukemia. (2006) 20:847–56. doi: 10.1038/sj.leu.2404132 16453000

[32] Windeløv NA Johansson PI Sørensen AM Perner A Wanscher M Larsen CF. Low level of procoagulant platelet microparticles is associated with impaired coagulation and transfusion requirements in trauma patients. J Trauma Acute Care Surg. (2014) 77:692–700. doi: 10.1097/TA.0000000000000437 25494419

[33] Henrich D. Focus on biomarkers, confounders and new therapeutic approaches in trauma. Eur J Trauma Emerg Surg. (2022) 48:1521–3. doi: 10.1007/s00068-022-01976-1 PMC9192362 35701902

[34] Sterner RC Sterner RM. Immune response following traumatic spinal cord injury: Pathophysiology and therapies. Front Immunol. (2023) 13:1084101. doi: 10.3389/fimmu.2022.1084101 36685598 PMC9853461

[35] Li C Wu Z Zhou L Shao J Hu X Xu W. Temporal and spatial cellular and molecular pathological alterations with single-cell resolution in the adult spinal cord after injury. Sig Transduct Target Ther. (2022) 7:65. doi: 10.1038/s41392-022-00885-4 PMC8888618 35232960

[36] Li R Ye JJ Gan L Zhang M Sun D Li Y. Traumatic inflammatory response: pathophysiological role and clinical value of cytokines. Eur J Trauma Emerg Surg. (2023) 50(4):1313–30. doi: 10.1007/s00068-023-02388-5 PMC11458723 38151578

[37] Hellenbrand DJ Quinn CM Piper ZJ Morehouse CN Fixel JA Hanna AS. Inflammation after spinal cord injury: a review of the critical timeline of signaling cues and cellular infiltration. J Neuroinflamm. (2021) 18:284. doi: 10.1186/s12974-021-02337-2 PMC8653609 34876174

[38] Kaur S Isenberg JS Roberts DD. CD47 (Cluster of differentiation 47). Atlas Genet Cytogenet Oncol Haematol. (2021) 25:83–102. 34707698 PMC8547767

[39] Barclay AN Van Den Berg TK. The interaction between signal regulatory protein alpha (SIRPα) and CD47: structure, function, and therapeutic target. Annu Rev Immunol. (2014) 32:25–50. doi: 10.1146/annurev-immunol-032713-120142 24215318

[40] Gitik M Liraz-Zaltsman S Oldenborg PA Reichert F Rotshenker S. Myelin down-regulates myelin phagocytosis by microglia and macrophages through interactions between CD47 on myelin and SIRPα (signal regulatory protein-α) on phagocytes. J Neuroinflamm. (2011) 8:24. doi: 10.1186/1742-2094-8-24 PMC3068094 21401967

[41] Gitik M Elberg G Reichert F Tal M Rotshenker S. Deletion of CD47 from Schwann cells and macrophages hastens myelin disruption/dismantling and scavenging in Schwann cells and augments myelin debris phagocytosis in macrophages. J Neuroinflamm. (2023) 20:243. doi: 10.1186/s12974-023-02929-0 PMC10594853 37872624

[42] Han MH Lundgren DH Jaiswal S Chao M Graham KL Garris CS. Janus-like opposing roles of CD47 in autoimmune brain inflammation in humans and mice. J Exp Med. (2012) 209:1325–34. doi: 10.1084/jem.20101974 PMC3405500 22734047

[43] Ben XY Wang YR Zheng HH Li DX Ren R Ni PL. Construction of exosomes that overexpress CD47 and evaluation of their immune escape. Front Bioeng Biotechnol. (2022) 10:936951. doi: 10.3389/fbioe.2022.936951 35845399 PMC9279928

[44] Zhuang W Li B. Suppression of extracellular vesicle CD47 induces systemic anti-DLBCL immunity. Blood. (2021) 138:716–6. doi: 10.1182/blood-2021-152451

[45] Van Acker HH Capsomidis A Smits EL Van Tendeloo VF. CD56 in the immune system: more than a marker for cytotoxicity? Front Immunol. (2017) 8:892. doi: 10.3389/fimmu.2017.00892 28791027 PMC5522883

[46] Xu L Zhang Y Zhang R Zhang H Song P Ma T. Elevated plasma BDNF levels are correlated with NK cell activation in patients with traumatic spinal cord injury. Int Immunopharmacol. (2019) :74:105722. doi: 10.1016/j.intimp.2019.105722 31255880

[47] Gunesch JT Dixon AL Ebrahim TA Berrien-Elliott MM Tatineni S Kumar T. CD56 regulates human NK cell cytotoxicity through Pyk2. eLife. (2020) 9:e57346. doi: 10.7554/eLife.57346 32510326 PMC7358009

[48] Martinez AL Shannon MJ Sloan T Mace EM. CD56/NCAM mediates cell migration of human NK cells by promoting integrin-mediated adhesion turnover. MBoC. (2024) 35:ar64. doi: 10.1091/mbc.E23-12-0463 38507235 PMC11151098

[49] Lugini L Cecchetti S Huber V Luciani F Macchia G Spadaro F. Immune surveillance properties of human NK cell-derived exosomes. J Immunol. (2012) 189:2833–42. doi: 10.4049/jimmunol.1101988 22904309

[50] Segura E Nicco C Lombard B Véron P Raposo G Batteux F. ICAM-1 on exosomes from mature dendritic cells is critical for efficient naive T-cell priming. Blood. (2005) 106:216–23. doi: 10.1182/blood-2005-01-0220 15790784

[51] Riegger T Conrad S Schluesener HJ Kaps HP Badke A Baron C. Immune depression syndrome following human spinal cord injury (SCI): A pilot study. Neuroscience. (2009) 158:1194–9. doi: 10.1016/j.neuroscience.2008.08.021 18790013

[52] Herman P Stein A Gibbs K Korsunsky I Gregersen P Bloom O. Persons with chronic spinal cord injury have decreased natural killer cell and increased toll-like receptor/inflammatory gene expression. J Neurotrauma. (2018) 35:1819–29. doi: 10.1089/neu.2017.5519 PMC6033303 29310515

[53] Rutishauser U Thiery JP Brackenbury R Edelman GM. Adhesion among neural cells of the chick embryo. III. Relationship of the surface molecule CAM to cell adhesion and the development of histotypic patterns. J Cell Biol. (1978) 79:371–81. doi: 10.1083/jcb.79.2.371 PMC2110256 569155

[54] Zhang S Xia YY Lim HC Tang FR Feng ZW. NCAM-mediated locomotor recovery from spinal cord contusion injury involves neuroprotection, axon regeneration, and synaptogenesis. Neurochem Int. (2010) 56:919–29. doi: 10.1016/j.neuint.2010.03.023 20381564

[55] Sun Y Liu Q Qin Y Xu Y Zhao J Xie Y. Exosomes derived from CD271 ^+^ CD56 ^+^ bone marrow mesenchymal stem cell subpopoulation identified by single-cell RNA sequencing promote axon regeneration after spinal cord injury. Theranostics. (2024) 14:510–27. doi: 10.7150/thno.89008 PMC10758065 38169566

[56] Betjes MGH Haks MC Tuk CW Beelen RHJ. Monoclonal antibody EBM11 (Anti-CD68) discriminates between dendritic cells and macrophages after short-term culture. Immunobiology. (1991) 183:79–87. doi: 10.1016/S0171-2985(11)80187-7 1834546

[57] Hendrickx DAE Van Eden CG Schuurman KG Hamann J Huitinga I. Staining of HLA-DR, Iba1 and CD68 in human microglia reveals partially overlapping expression depending on cellular morphology and pathology. J Neuroimmunol. (2017) 309:12–22. doi: 10.1016/j.jneuroim.2017.04.007 28601280

[58] Winston CN Sarsoza F Spencer B Rissman RA. Characterizing blood-based, microglial derived exosomes (MDEs) as biomarkers for Alzheimer’s disease. Alzheimer’s Dementia. (2021) 17:e055371. doi: 10.1002/alz.055371

[59] Colonna M Butovsky O. Microglia function in the central nervous system during health and neurodegeneration. Annu Rev Immunol. (2017) 35:441–68. doi: 10.1146/annurev-immunol-051116-052358 PMC8167938 28226226

[60] Van Den Broek B Pintelon I Hamad I Kessels S Haidar M Hellings N. Microglial derived extracellular vesicles activate autophagy and mediate multi-target signaling to maintain cellular homeostasis. J Extracell Vesicle. (2020) 10:e12022. doi: 10.1002/jev2.12022 PMC7890546 33708355

[61] Lombardi M Parolisi R Scaroni F Bonfanti E Gualerzi A Gabrielli M. Detrimental and protective action of microglial extracellular vesicles on myelin lesions: astrocyte involvement in remyelination failure. Acta Neuropathol. (2019) 138:987–1012. doi: 10.1007/s00401-019-02049-1 31363836 PMC6851224

[62] Kwiecien JM Dabrowski W Dąbrowska-Bouta B Sulkowski G Oakden W Kwiecien-Delaney CJ. Prolonged inflammation leads to ongoing damage after spinal cord injury. PloS One. (2020) 15:e0226584. doi: 10.1371/journal.pone.0226584 32191733 PMC7081990

[63] Almahmoud K Namas RA Abdul-Malak O Zaaqoq AM Zamora R Zuckerbraun BS. Impact of injury severity on dynamic inflammation networks following blunt trauma. Shock. (2015) 44:101–9. doi: 10.1097/SHK.0000000000000395 PMC4504837 26009819

[64] Calligaris M Cuffaro D Bonelli S Spanò DP Rossello A Nuti E. Strategies to target ADAM17 in disease: from its discovery to the iRhom revolution. Molecules. (2021) 26:944. doi: 10.3390/molecules26040944 33579029 PMC7916773

[65] Wei Z Yu D Bi Y Cao Y disintegrin A. and metalloprotease 17 promotes microglial cell survival via epidermal growth factor receptor signalling following spinal cord injury. Mol Med Rep. (2015) 12:63–70. doi: 10.3892/mmr.2015.3395 25738567 PMC4438914

[66] Vidal PM Lemmens E Avila A Vangansewinkel T Chalaris A Rose-John S. ADAM17 is a survival factor for microglial cells in *vitro* and in *vivo* after spinal cord injury in mice. Cell Death Dis. (2013) 4:e954–4. doi: 10.1038/cddis.2013.466 PMC3877539 24336074

[67] Barros CS Nguyen T Spencer KSR Nishiyama A Colognato H Müller U. [amp]]beta;1 integrins are required for normal CNS myelination and promote AKT-dependent myelin outgrowth. Development. (2009) 136:2717–24. doi: 10.1242/dev.038679 PMC2730401 19633169

[68] Chavaroche A Cudic M Giulianotti M Houghten RA Fields GB Minond D. Glycosylation of a disintegrin and metalloprotease 17 affects its activity and inhibition. Anal Biochem. (2014) 449:68–75. doi: 10.1016/j.ab.2013.12.018 24361716 PMC4334441

[69] Cardeñes B Clares I Toribio V Pascual L López-Martín S Torres-Gomez A. Cellular integrin α5β1 and exosomal ADAM17 mediate the binding and uptake of exosomes produced by colorectal carcinoma cells. IJMS. (2021) 22:9938. doi: 10.3390/ijms22189938 34576100 PMC8471098

[70] Gooz P Dang Y Higashiyama S Twal WO Haycraft CJ Gooz M. A disintegrin and metalloenzyme (ADAM) 17 activation is regulated by α5β1 integrin in kidney mesangial cells. PloS One. (2012) 7:e33350. doi: 10.1371/journal.pone.0033350 22413019 PMC3297637

[71] Hardy BM King KL Enninghorst N Balogh ZJ. Trends in polytrauma incidence among major trauma admissions. Eur J Trauma Emerg Surg. (2022) 50(3):623–6. doi: 10.1007/s00068-022-02200-w PMC11249606 36536173

[72] Pape M Giannakópoulos GF Zuidema WP De Lange-Klerk ESM Toor EJ Edwards MJR. Is there an association between female gender and outcome in severe trauma? A multi-center analysis in the Netherlands. Scand J Trauma Resusc Emerg Med. (2019) 27:16. doi: 10.1186/s13049-019-0589-3 30760289 PMC6373135

