# Supplementary material for: Extracellular vesicles epitopes as potential biomarker candidates in patients with traumatic spinal cord injury
Source: Front Immunol. 2024 Nov 27;15:1478786. doi: 10.3389/fimmu.2024.1478786 (PMC11656158; doi:10.3389/fimmu.2024.1478786)
Supplement: Supplementary file 1 [file DataSheet1.docx]

**Supplementary appendix**

**Table S1. MACSPlex EV Prototype Kit Neuro-beads populations.**

|  | **Antibody** | **Bead No.** | **Name** | **Isotyp** |
| --- | --- | --- | --- | --- |
| Panel A | CD68 | 22 | CD68 | recombinant human IgG1 |
| Panel A | CD340 | 23 | CD340 | mouse IgG1k |
| Panel A | CD49a | 24 | CD49a | recombinant human IgG1 |
| Panel A | CD140a | 32 | CD140a | recombinant human IgG1 |
| Panel A | CD171 | 33 | CD171 | recombinant human IgG1 |
| Panel A | CD56 | 34 | CD56 | recombinant human IgG1 |
| Panel A | CD13 | 35 | CD13 | recombinant human IgG1 |
| Panel A | BDNF | 42 | Brain-derived neurotrophic factor | recombinant human IgG1 |
| Panel A | CD31 | 43 | CD31 | recombinant human IgG1 |
| Panel A | GFAP | 44 | Glial fibrillary acidic protein | recombinant human IgG1 |
| Panel A | CD222 | 45 | CD222(aIGF2R) | recombinant human IgG1 |
| Panel A | CD11b | 46 | CD11b | rat IgG2bκ |
| Panel A | CD24 | 52 | CD24 | recombinant human IgG1 |
| Panel A | CD45 | 53 | CD45 | recombinant human IgG1 |
| Panel A | CD133 | 54 | CD133/1 | recombinant human IgG1 |
| Panel A | O4 | 55 | O4 | mouse IgM |
| Panel A | Glast | 56 | Glutamate transporter Glast | mouse IgG2aκ |
| Panel A | CD90 | 57 | CD90 | recombinant human IgG1 |
| Panel A | CX3CR1 | 63 | Chemokine Receptor CX3CR1 | recombinant human IgG1 |
| Panel A | EGF | 64 | Epidermal growth factor | recombinant human IgG1 |
| Panel A | CD325 | 65 | CD325 | mouse IgG1κ |
| Panel A | Podoplanin | 66 | Podoplanin | recombinant human IgG1 |
| Panel A | A2b5 | 67 | A2B5 | mouse IgMκ |
| Panel A | CD29 | 68 | CD29 | recombinant human IgG1 |
| Panel A | CSPG4 | 74 | Chondroitin sulphate proteoglycan 4 (AN2) | rat IgG1 |
| Panel A | CD9 | 75 | CD9 | mouse IgG1 |
| Panel A | CD63 | 76 | CD63 | mouse IgG1κ |
| Panel A | CD81 | 77 | CD81 | recombinant human IgG1 |
| Panel A | CD47 | 78 | CD47 | recombinant human IgG1 |
| Panel A | PSA-Ncam | 79 | PSA-Neuron Cell Adhesion Molecule | mouse IgM |
| Panel A | CD44 | 85 | CD44 | mouse IgG1κ |
| Panel A | CD38 | 86 | CD38 | recombinant human IgG1 |
| Panel A | CD49f | 87 | CD49f | recombinant human IgG1 |
| Panel A | CD106 | 88 | CD106 | recombinant human IgG1 |
| Panel A | CD119 | 89 | CD119 | recombinant human IgG1 |
| Panel A | CD49e | 96 | CD49e | recombinant human IgG1 |
| Panel A | CD54 | 97 | CD54 | recombinant human IgG1 |
| Panel A | CD36 | 98 | CD36 | recombinant human IgG1 |
| Panel A | CD45RB | 99 | CD45RB | recombinant human IgG1 |
| Panel B | CD18 | 22 | CD18 | recombinant human IgG1 |
| Panel B | CD64 | 23 | CD64 | recombinant human IgG1 |
| Panel B | CD107a | 24 | CD107 | mouse IgG1κ |
| Panel B | CD180 | 32 | CD180 | recombinant human IgG1 |
| Panel B | CD196 | 33 | CD196 | recombinant human IgG1 |
| Panel B | ADAM17 | 34 | A disintegrin and metalloprotease 17 | mouse IgG2bκ |
| Panel B | APP | 35 | Amyloid-beta precursor protein | mouse IgG1κ |
| Panel B | CLDN5 | 42 | Claudin-5 | mouse IgG2bκ |
| Panel B | ENO2 | 43 | Enolase 2 (neuron specific enolase) | mouse IgG2b |
| Panel B | GALC | 44 | Galactosylceramidase | moues IgG2aκ |
| Panel B | Tau | 56 | Tau | recombinant human IgG1 |
| Panel B | PLP_2 | 57 | Proteolipid Protein 2 | recombinant human IgG1 |
| Panel B | MBP | 63 | Myelin Basic Protein | recombinant human IgG1 |
| Panel B | VGlut2 | 64 | Vesicular glutamate transporter 2 | recombinant human IgG1 |
| Panel B | GD-2 | 78 | Ganglioside GD 2 | recombinant human IgG1 |
| Panel B | TH | 76 | Tyrosine hydroxylase | recombinant human IgG1 |
| Panel B | CD146 | 86 | CD146 | recombinant human IgG1 |
| Panel B | CD11b (REA) | 87 | CD11b | recombinant human IgG1 |
| Panel B | NEFH | 96 | Neurofilament Heavy Chain | recombinant human IgG1 |
| Panel B | PVALB | 97 | Parvalbumin | recombinant human IgG1 |
| Panel B | TUBB3 | 98 | Tubulin beta-3 chain | recombinant human IgG1 |
| Panel B | SYP | 99 | Synaptophysin | mouse IgG1 |
| Panel A/B | REA control | 77 | REA control | recombinant human IgG1 |
| Panel A/B | mIgG1 control | 79 | mIgG1 control | mouse IgG1 |


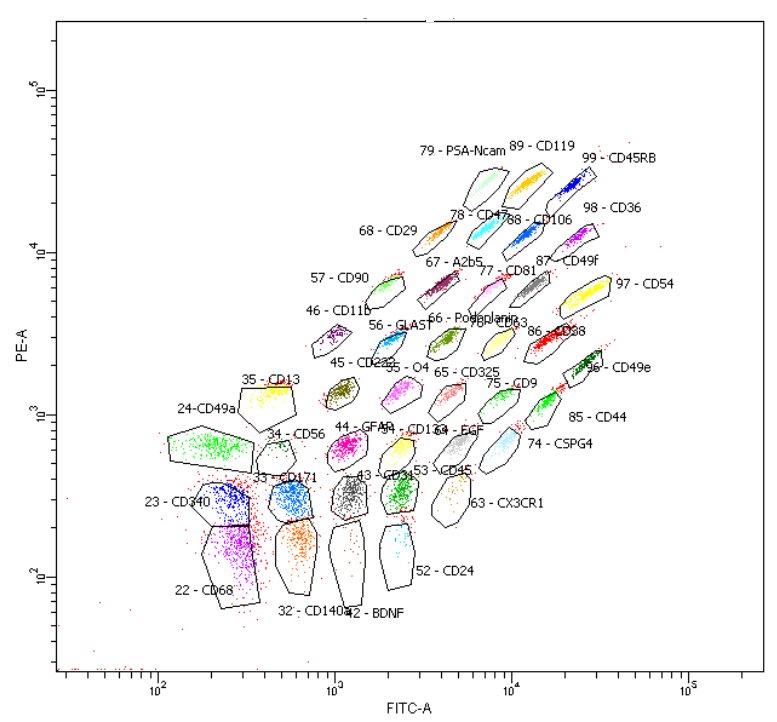


**Figure S1. Detection of MACSPlex EV Capture Bead populations in a MACSPlex-FITC versus MACSPlex-PE dot plot; representative image.**

**
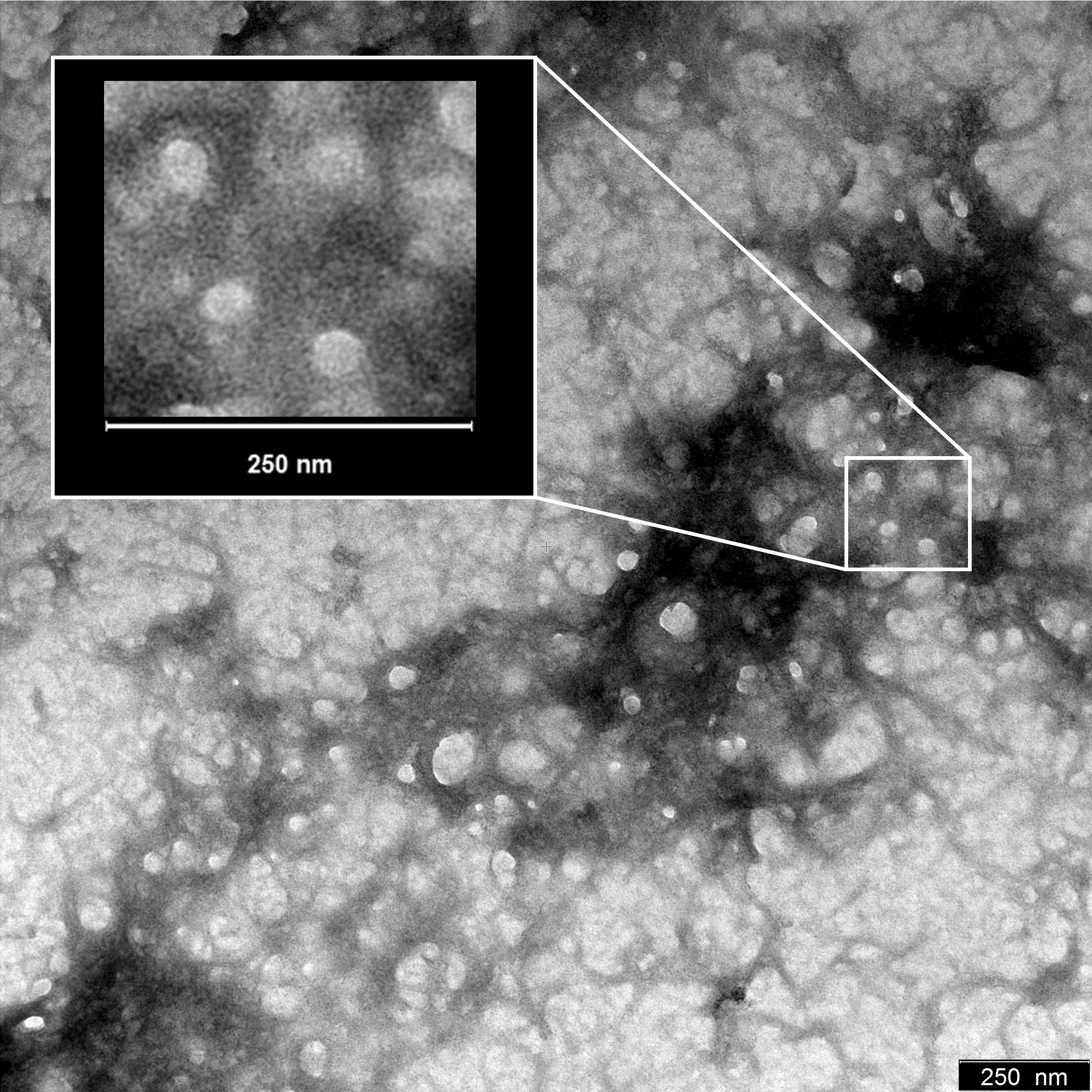
**

**Figure S2. Transmission electron microscopy of the EV isolate (healthy volunteer), raw image.** Image was captured using a wide-angle 2K-CCD-CKamera (TRS) and a Zeiss EM900 TEM. ImageSP Software for Transmisson Electron Microscopy (SYSPROG).
